# Influence of different post-polymerization methods on the dimensional accuracy of additively manufactured dental crowns

**DOI:** 10.2340/biid.v12.45100

**Published:** 2025-12-30

**Authors:** Jason Cao, Xiaoyun Liu, Andrew B. Cameron, John Aarts, Joanne Jung Eun Choi

**Affiliations:** a Sir John Walsh Research Institute, Faculty of Dentistry, University of Otago, Dunedin, New Zealand; b Faculty of Dentistry, Griffith University, Gold Coast, Australia

**Keywords:** Additive manufacturing, 3D-printing, post-polymerization, dimensional accuracy, dental crowns

## Abstract

**Purpose:**

To evaluate the influence of different post-polymerization methods and types of polymerization units on the dimensional accuracy of additive manufactured (AM) permanent crown resins.

**Methods:**

Three crown materials were evaluated: two permanent restoration-indicated resins (Saremco print Crowntec and Varseosmile Crown^plus^) and one long-term temporary resin (NextDent C&B MFH). These were additively manufactured and post-polymerized using four different polymerization units with varying protocols. Post-polymerization was performed either at room temperature or at 60°C, as well as without an inert medium, with glycerine, or with nitrogen as the inert medium. A typical mandibular molar full crown design was used and subtractively manufactured (SM) crowns served as the control group. Post-processed AM crowns were digitally scanned, and three specific regions (margin, intaglio surface and interproximal contacts) of the STL of the crowns were analyzed using Geomagic Control X software. Root Mean Square (RMS) and average deviation values were collected and statistically analyzed in PRISM version 9. Additionally, scanning electron microscopy analysis was conducted.

**Results:**

The SM crowns exhibited the highest accuracy (the lowest RMS) for both the intaglio (18.6 ± 0.9 µm) and marginal surfaces (32.5 ± 3 µm). AM crowns showed variable accuracy depending on the post-polymerization method. Crowns post-polymerized in glycerine generally demonstrated lower RMS values. The glass filler-reinforced resin composite (Crowntec) material consistently exhibited higher RMS values (89 ± 5.4 µm to 120 ± 8.1 µm) across all regions studied. Post-polymerization temperatures did not significantly impact the dimensional accuracy of any of the materials studied (*p* > 0.05).

**Conclusion:**

Using glycerine during post-polymerization, alongside the manufacturer’s recommended polymerization unit, generally enhanced the dimensional accuracy of AM dental crowns. SM crowns demonstrated superior accuracy in the intaglio and marginal surfaces; however, no significant differences were observed in the interproximal compared to AM crowns.

**Clinical significance:**

Given the variety of available post-polymerization units and protocols, dental practitioners should consider their impact on the dimensional accuracy of AM dental crowns. Awareness of these effects can help to prevent issues with crown accuracy, including adaptation to preparations and the contact points. Furthermore, this study provides alternative post-polymerization methods for practitioners lacking access to the manufacturer’s recommended equipment.

## Introduction

Computer-aided design and computer-aided manufacturing (CAD/CAM) have become established technologies in modern dentistry [1]. Additive and subtractive manufacturing methods are currently utilized to produce accurate restorations with functionally aesthetic morphology generated by CAD software [2]. Additive manufacturing (AM), also known as 3D printing, has grown increasingly popular in the past decade [3]. Stereolithography (SLA) and digital light processing (DLP) are common AM technologies used in digital dentistry to create a wide range of appliances, including dental models, crown and bridge restorations, and dentures [4–6]. These technologies use photopolymer AM resins, constructed layer-by-layer using either a UV laser for SLA [7] or a light projector for DLP [8]. Post-processing is a crucial step in these techniques. It involves post-process washing to remove excess photopolymer in a solvent, followed by post-polymerization with additional light exposure under specific parameters [3]. A variety of post-processing equipment and products are used, following each material’s manufacturer’s instructions for use (IFU), such as the proper solutions, exposure times, and consistent washing will produce consistent post-polymerization results. Deviations from the IFU can negatively impact the dimensional accuracy, biocompatibility, and mechanical properties of the material [9]. Some post-polymerization units use varying temperatures to assist in post-polymerizing AM objects. Temperature has been shown to influence the mechanical properties and accuracy of AM materials, with higher post-polymerization temperatures reducing the duration of post-polymerization while improving the mechanical properties and degree of conversion of the AM resin [10].

Subtractive manufacturing (SM) is an alternative method used in digital dentistry, where restorations are milled from densely or partially sintered ceramic blocks using various types of burs. This method produces more waste than AM, as it requires the removal of large amounts of material. The nature of SM can also lead to chipping, surface flaws, and micro-cracking, especially in fully sintered or crystalized blocks, affecting the fit of restorations and weakening their mechanical properties [11]. Despite these limitations, SM remains widely used in dentistry due to its superior mechanical properties and biocompatibility compared to AM materials [12].

The marginal integrity and the accuracy of the intaglio fit of crown and bridge restorations are crucial for both AM and SM to ensure the longevity of the restoration [13]. Inaccurately manufactured restorations can lead to issues such as secondary caries, marginal micro-leakage, and plaque accumulation on indirect restorations [14]. Therefore, the dimensional accuracy of dental restorations must be carefully considered during the manufacturing process. Accuracy consists of trueness and precision. Trueness describes how closely the manufactured model matches the reference, while precision refers to the consistency between multiple samples. The use of 3D analytical software, referred to as metrology software, to measure and analyze accuracy across various surfaces has become the gold standard [15]. The outcomes of this type of assessment are often negative deviations, positive deviations, root mean square estimate (RMS) [16]. RMS describes the typical size of a set of values, by measuring the error between predicted and actual values. It was suggested that including positive and negative mean deviation values should be utilized alongside RMS values to provide a more comprehensive interpretation of the data [17].

To date, no research has been conducted on how the specifications of different polymerization units affect the dimensional accuracy of various AM resin crown materials. Therefore, this study aims to investigate different post-polymerization methods and temperatures and their effects on the dimensional accuracy of AM resins for dental crown applications, compared to an SM group as a control. The null hypothesis is that different post-polymerization conditions (use of inert medium of glycerine or nitrogen) and temperatures (room temperature and 60°C) will not influence the dimensional accuracy of AM dental crown materials.

## Materials and methods

### Materials used and specimen preparation

**Figure 1:**
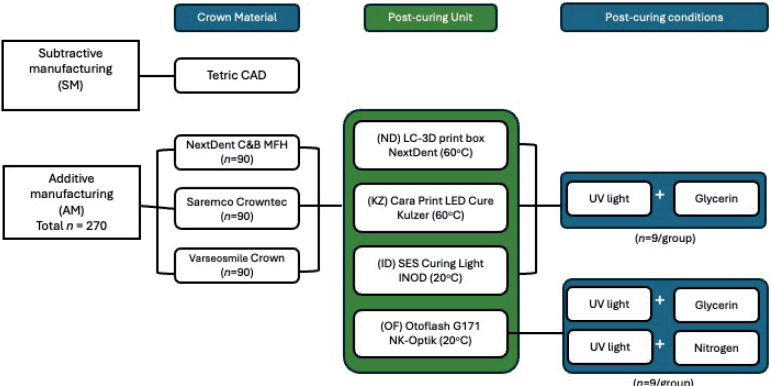
Study design flow diagram.

A mandibular left first molar (Tooth 36) crown was prepared and designed on a typodont plastic tooth (NISSIN, Japan) with shoulder margins to simulate a typical single crown dental restoration. The crown design (Dental System, 3Shape, Copenhagen) included a milling compensation of 0.6 mm and was exported as a standard tessellation language file. To fabricate the crowns, three AM crown and bridge resin materials and one polymethyl methacrylate (PMMA) material for SM crowns were used (Table 1). A sample size calculation using G. power (Universitat Dusseldolf) was conducted based on the preliminary pilot run results. An a priori power analysis (omnibus between-groups comparsion (fixed effects), *a* = 0.05, power = 0.95 m effect size *f* = 0.5 and power (1-β) = 0.95) indicated that a sample size of nine specimens per group (*n* = 9) would provide a statistical power of 95. A total of 270 AM crowns (*N* = 90/material type) and 9 SM crown specimens were manufactured as shown in Figure 1.

**Table 1:** Description of the materials used in the experiment.

Product name	Manufacturer	Composition
Tetric CAD	Ivoclar Vivadent	Resin Composite (% not published)
NextDent C&B MFH	NextDent 3D Systems	Methacrylic oligomer (>60 % w/w), glycol methacrylate (15–25% w/w), phosphine oxide (<2.5% w/w)
Varseosmile Crown ^plus^	Bego	Esterification products of 4.4; isopropylidenediphenol, ethoxylated and 2-methylprop-2-enoic acid (50.00 – <75.00 wt%), diphenyl(2,4,6,-trimethylbenzoyl)phosphine oxide (<2.5 wt%)
Crowntec	Saremco	BisEMA (50 – <70%), Trimethylbenzonyldiphenylphosphine oxide (0.1 – <1%)

### Crown manufacturing and post-polymerization process

Ten composite resin crowns (Tetric CAD, Ivoclar Vivadent) were milled using an SM machine (PrograMill PM7, Ivoclar) to serve as the control group. AM crowns were printed using a DLP printer (Asiga Max UV; Asiga) with a layer thickness of 50 µm, and each print cycle produced 45 crowns. There is no clear consensus on the most accurate printing orientation for dental crowns [18]. As a result, a printing orientation of 45 degrees was chosen so that there was consistency across the groups during manufacturing to ensure this was not a variable that would affect the results. Supports were duplicated and attached to the occlusal and buccal surfaces of each crown, as these areas are not clinically significant. The same bottle, and subsequently batch number, of each material was used to avoid contamination and ensure a consistent and homogenous mix of the material.

AM crowns made from the hybrid resin composites (Varseosmile Crown^plus^ and NextDent C&B MFH) crowns were rinsed in 99.5% ethanol for 3 min, followed by a 2-min rinse in clean ethanol, and then air dried according to the manufacturer’s instructions. Glass filler-reinforced resin composite (Saremco Crowntec) crowns were cleaned using an alcohol-soaked brush, as recommended, and kept in the dark environment before and after cleaning to minimize unwanted polymerization. Support structures were removed with a scalpel and each crown was visually inspected for defects then post-polymerized using a specific unit and method.

Four types of polymerization units were used: Cara Print LEDcure (Kulzer), Otoflash G171 (NK-Optik), LC-3DPrint Box (3D systems), and SES Curing Light (INNO DENTAL). Only the SES Curing Light acted as a conventional non-dental polymerization unit. Each unit’s polymerization time was 30 min, according to the material manufacturers’ recommendations, except for the Otoflash G171, which used 2,000 × 2 flashes (Table 2). For each material group, post-processing parameters followed each manufacturer’s IFU, with any deviations reported explicitly. Where a commonly adopted laboratory setting differed from an IFU (e.g. Otoflash 2,000×2 flashes for Varseosmile), we included this setting alongside IFU-aligned conditions for transparency and comparability.

**Table 2:** Characteristics of post-polymerization units and the conditions used.

Polymerization units	Manufacturer	Spectral distribution	Temperature (°C)	Polymerization duration	Post-polymerization conditions (crown number per material)
LC-3DPrint Box	NextDent	300–550 nm	60	30 min	UV light (*n* = 10)
					UV light and glycerine (*n* = 10)
Cara Print LEDCure	Kulzer	370–470 nm	60	30 min	UV light (*n* = 10)
					UV light and glycerine (*n* = 10)
Otoflash G171	NK-Optik	300–700 nm	Room Temperature	2000 × 2 flashes	UV light (*n* = 10)
					UV light and glycerine (*n* = 10)
					UV light and nitrogen (*n* = 10)
SES Curing Light	INNO DENTAL	LED, N/A	Room Temperature	30 min	UV light (*n* = 10)
					UV light and glycerine (*n* = 10)

Three main post-polymerization conditions were involved: (1) with ultra-violet (UV) light, (2) with UV light and glycerine liquid (Home Essentials Glycerol BP 100%) applied, and (3) with UV light and nitrogen. Nitrogen was used exclusively for the Otoflash polymerization unit as the three remaining units did not have this feature (Table 2 and Figure 1). This resulted in nine subgroups with 10 samples in each. Both nitrogen and glycerine are commonly used as an inert medium to prevent an oxygen inhibition layer from forming during post-polymerization [9, 19]. Crowns post-polymerized in glycerine were submerged in a glass disk filled with glycerine, and air bubbles were minimized to prevent the floating of the crowns. Post-polymerized crowns were stored in a dark environment for at least 24 h to ensure full polymerization and allow any material relaxation to occur.

### 3D analysis for accuracy comparisons

**Figure 2:**
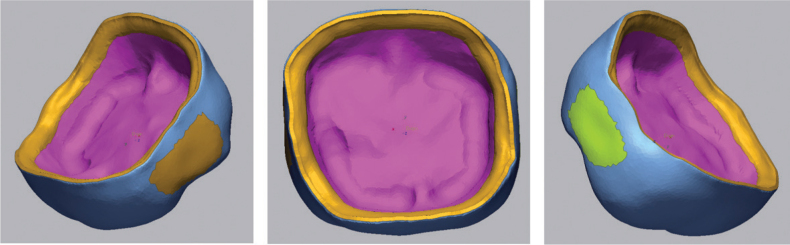
Segmented regions of the crown showing the distinct separation of the internal, marginal, mesial contacts and distal contacts.

Crowns were positioned on an AM custom-designed positioning jig, and scanned using a desktop optical scanner (E4, 3Shape, Copenhagen) and exported as standard tessellation language files. The original CAD design of the crown served as the ‘reference data’ and was superimposed with teach crown scan using ‘Precise Initial alignment’ followed by ‘Best Fit Alignment’. The intaglio, marginal, and interproximal contact areas of the master crown were segmented into a fixed region using Geomagic Control X software, as shown in Figure 2. This allowed for analysis in these individual regions Specific The ‘3D Compare’ process was used to analyze each fixed region per crown. RMS, negative deviations and positive deviation values were generated to represent changes from the master crown and scanned crown. The mesial and distal interproximal contacts were analyzed separately, generating two results per crown. These were later combined to represent the ‘interproximal contact’ surface. Mean deviations (µm) were also collected that represent the arithmetic mean of point-wise distances where negative values indicate contraction of materials and positive values represent expansion of materials. A color deviation map was also generated to visually display the discrepancies of the segmented regions to allow descriptive and visual analysis. Deviation maps were generated on a fixed ±120 µm scale and a ±30 µm tolerance band was applied to contextualize magnitude during qualitative interpretation.

### Scanning electron microscope analysis

One representative crown specimen from each test group was analyzed under scanning electron microscope (SEM). Ten specimens were mounted on aluminium stubs with double-sided carbon tape and carbon paste and coated with approximately 15 nm of gold palladium (Quorum Q150V Plus modular coating system, Quorum Technologies Limited, East Sussex, UK). Samples were viewed and imaged in a Zeiss Sigma 300VP FESEM (Carl Zeiss Inc, Oberkocken, Germany) at an accelerating voltage of 5 KV. Three areas were examined with the SEM: the margin, the flat intaglio surface, and the axial wall. All images were taken at consistent magnifications of 40x, 100x and 200x. Specific surfaces could not be consistently examined due to placement limitations within the SEM, but areas were carefully selected to closely match other examined regions.

### Statistical analysis

Statistical analysis was performed using PRISM Version 9 (GraphPad). Normality test was assessed via Shapiro–Wilk test and the homoscedasticity was assessed using Brown–Forsythe test. Distributional assumptions were assessed prior to inference. Analyses were stratified by materials. Within each group, subgroup comparisons were performed (type of curing unit, post-curing condition and temperature) using Kruskal–Wallis with Dunn’s post hoc (Bonferroni). Where a unit is limited to certain conditions (e.g. Otoflash only has the option of nitrogen, no ability to adjust the curing temperature), these subgroups were not included in comparisons (e.g. temperature effect was evaluated only in curing units that had options to adjust between room temperature and 60 degrees (LC-3D print, Cara LED Cure). Descriptive statistics were collected by the mean ± standard deviation (SD) to facilitate comparisons and rank-based inference remained unaffected by the choice of descriptive summary. The significance level was set at α = 0.05.

## Results

### Dimensional accuracy of the internal surfaces

**Figure 3:**
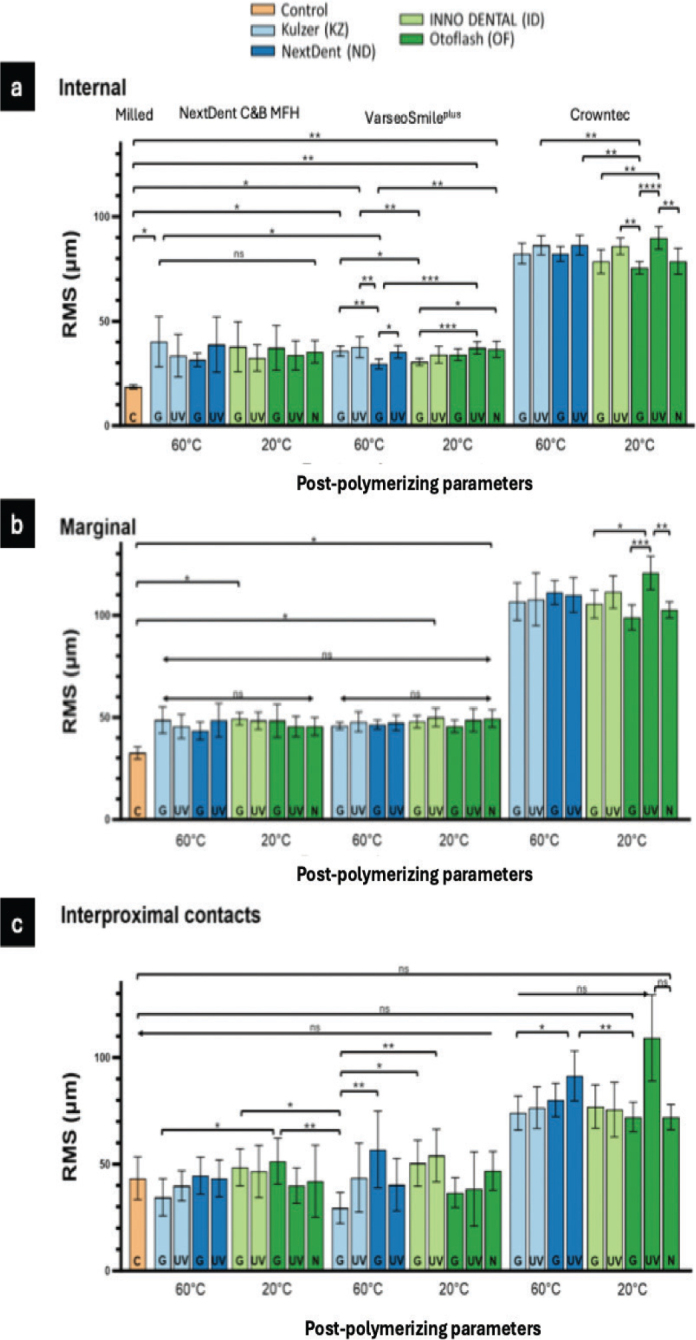
Root mean square (RMS) values of the materials internal (a), marginal surfaces (b) and interproximal surface (c) after post-polymerization with different parameters.

**Figure 4:**
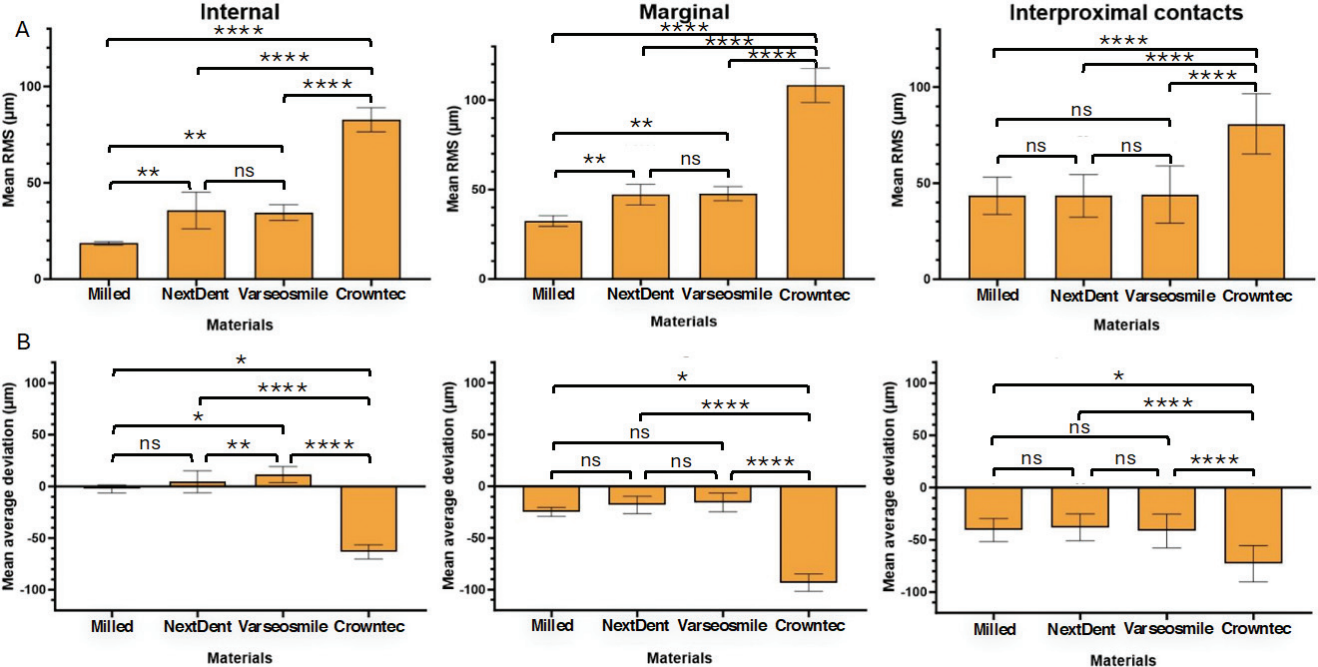
(A) Three graphs representing the mean RMS values of the materials’ internal, marginal, and interproximal surfaces. (B) Three graphs representing the mean positive and negative average deviation of the materials’ internal, marginal, and interproximal surfaces.

**Figure 6:**
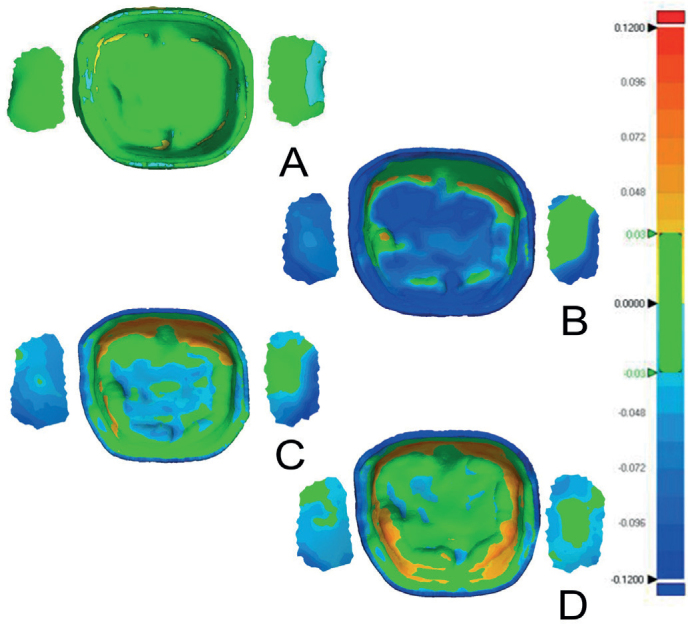
Color deviation maps of the intaglio surface and contact points for subtractively manufactured (A), Crowntec in Nitrogen (B), NextDent MFH in Nitrogen (C), Varseosmile in Nitrogen (D).

**Figure 7:**
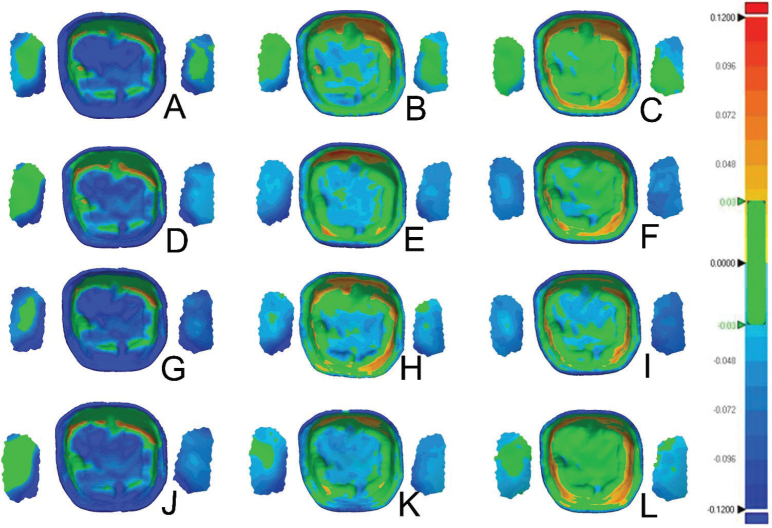
Color deviations maps of the intaglio surface and contact points for samples polymerized in glycerine for Crowntec (A, D, G, J), NextDent MFH (B, E, H, K), Varseosmile (C, F, I, L); Color deviation maps for processing units Cara Print LEDcure (A, B, C), SES Curing Light (D, E, F), LC-3DPrint Box (G, H, I), Otoflash G171 (J, K, L).

**Figure 8:**
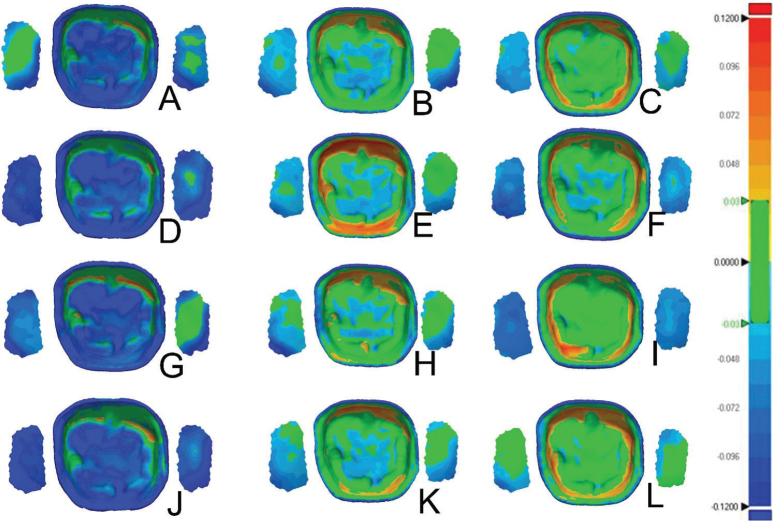
Color deviations maps of the intaglio surface and contact points for samples not polymerized in glycerine for Crowntec (A, D, G, J), NextDent MFH (B, E, H, K), Varseosmile (C, F, I, L); Color deviation maps for processing methods Cara Print LEDcure (A, B, C), SES Curing Light (D, E, F), LC-3DPrint Box (G, H, I), Otoflash G171 (J, K, L).

The dimensional accuracy analysis was calculated to produce the mean and standard deviation values for the RMS and the average positive and negative deviations (Tables 3–5 and Figures 3–4). Color deviation graphics were generated that show the trueness for all the test groups (Figures 6–8). This analysis was performed to assess the impact of various post-polymerization methods and units on the intaglio, marginal, and interproximal surfaces of the crowns.

**Table 3:** The mean RMS ± SD (µm) and mean positive and negative deviation ± SD (µm) of the four tested materials.

Materials		Tetric CAD (subtractively manufactured from PrograMill PM7)	NextDent C&B MFH	Varseosmile Crown^plus^	Crowntec
Mean RMS values ± SD					
Surfaces	Internal	18.99 ± 0.96	35.63 ± 9.481	34.55 ± 4.10	82.75 ± 6.30
	Marginal	32.49 ± 2.97	47.10 ± 5.81	47.69 ± 4.01	108.3 ± 9.62
	Interproximal contacts	23.26 ± 5.76	43.43 ± 11.02	44.10 ± 14.87	80.89 ± 15.67
Mean positive and negative deviation ± SD					
Surfaces	Internal	−9.82 ± 2.21	4.56 ± 10.60	11.52 ± 7.68	−63.33 ± 6.91
	Marginal	−17.42 ± 1.13	−18.07 ± 8.36	−15.62 ± 9.17	−93.10 ± 8.39
	Interproximal contacts	−19.11 ± 7.13	−38.01 ± 12.79	−41.61 ± 16.00	−72.95 ± 17.25

RMS: root mean square; SD: standard deviation.

**Table 5:** Mean ± standard deviation (µm) of the average positive and negative deviation values for the internal, marginal, and the average mesial and distal surfaces of specimens post-polymerization with different methods and units.

Post-polymerization unit		Kulzer		NextDent		INNO DENTAL		Otoflash		
		Glycerine	UV	Glycerine	UV	Glycerine	UV	Glycerine	UV	Nitrogen
**Internal surface**										
**Material**	NextDent C&B MFH	9.3 ± 15	4.4 ± 10.1	−1.2 ± 6.4	9.5 ± 11.7	2.7 ± 10.6	4.1 ± 7.6	−3.8 ± 8	6.1 ± 7.9	10.1 ± 10
	Varseosmile Crown^Plus^	19.6 ± 3.6	12.2 ± 8.7	2.8 ± 4.9	11.6 ± 8	6.4 ± 6.9	8.7 ± 5.8	15 ± 2.7	15.9 ± 7.4	11.5 ± 5.6
	Crowntec	−61.9 ± 4.7	−67.3 ± 4.4	−61.7 ± 3.8	−70.3 ± 4.7	−59.8 ± 6.5	−65.1 ± 3.6	−55.8 ± 3.2	−70.3 ± 6.2	−57.8 ± 6.7
**Marginal surface**										
**Material**	NextDent C&B MFH	−15.7 ± 8.1	−19.8 ± 4.7	−19.7 ± 7.1	−13.4 ± 10.1	−21.9 ± 6.7	−20.5 ± 10.7	−22.3 ± 8.7	−14.5 ± 6.3	−14.9 ± 8.4
	Varseosmile Crown^Plus^	−9.2 ± 3.9	−14.3 ± 12.5	−19 ± 12.3	−12.2 ± 6.8	−21.5 ± 7.6	−21 ± 6	−12.2 ± 6.5	−14.6 ± 11	−16.6 ± 7.3
	Crowntec	−91.9 ± 9	−93.3 ± 9.8	−96.1 ± 5.6	−95.2 ± 6.5	−93.2 ± 7.3	−95.1 ± 3.6	−82.4 ± 6.5	−102.8 ± 6.1	−88 ± 4.1
**Average mesial and distal surfaces**										
**Material**	NextDent C&B MFH	−27.9 ± 9.9	−33 ± 9.3	−40 ± 9	−38.8 ± 10	−44.5 ± 10.7	−41.3 ± 14.7	−46.9 ± 11.4	−34.4 ± 10.6	−35.9 ± 19.2
	Varseosmile Crown^Plus^	−25.5 ± 8.6	−40.6 ± 17.8	−55.1 ± 19.3	−37.6 ± 13.1	−48.7 ± 10.9	−52.1 ± 13.6	−34.2 ± 8.3	−35.6 ± 18.6	−45.2 ± 9.4
	Crowntec	−65.7 ± 8.8	−68.9 ± 12.1	−72.6 ± 8.3	−84.9 ± 13.1	−66.4 ± 12.1	−68.4 ± 14.4	−62.4 ± 7.6	−103.8 ± 20.8	−63.4 ± 6.8

Both the intaglio and marginal surface of SM crowns (control group) had the lowest RMS values (intaglio = 18.9 ± 0.9 µm, marginal = 32.5 ± 3 µm), suggesting that they are the most dimensionally accurate compared to the AM crowns (Table 3, Figures 3 and 4). The color deviation map of the control group showed minimal surface deviations across all examined areas (Figure 6A). In terms of all AM crowns, the RMS values of their intaglio and marginal surface exhibited variations based on the post-polymerization conditions and units employed.

NextDent C&B MFH (AM-ND) crowns exhibited the highest RMS values for intaglio and marginal surfaces when post-polymerized with glycerine in the Kulzer (KZ) (40.2 ± 12 µm) and Inno Dental (ID) units (49.4 ± 3.1 µm), respectively. The lowest values for both regions were observed with the ND post-polymerization unit using glycerine (Table 4). No statistically significant differences were found across post-polymerization conditions for both the intaglio and marginal surfaces within the AM-ND groups.

**Table 4:** The mean RMS values ± SD (µm) of AM crowns after post-polymerization with different methods and units.

Post-polymerization unit		Kulzer		NextDent		INNO DENTAL		Otoflash		
		Glycerine	UV	Glycerine	UV	Glycerine	UV	Glycerine	UV	Nitrogen
**Internal surface**										
**Material**	NextDent C&B MFH	40.2 ± 12	33.6 ± 10.2	31.5 ± 3.2	38.9 ± 13.2	37.8 ± 11.9	32.4 ± 6.29	37.3 ± 10.8	33.7 ± 7	35.4 ± 5.3
	Varseosmile Crown^Plus^	35.7 ± 2.5	37.6 ± 5	29.6 ± 2.4	35.4 ± 2.9	30.8 ± 1.9	34 ± 4.1	34 ± 2.8	37.3 ± 3	36.6 ± 3.8
	Crowntec	82.4 ± 4.9	86.3 ± 4.6	82.1 ± 3.5	86.4 ± 4.6	78.5 ± 5.7	85.2 ± 4.1	75.5 ± 3	89.8 ± 5.4	78.6 ± 6.2
**Marginal surface**										
**Material**	NextDent C&B MFH	48.7 ± 6.4	45.7 ± 5.9	43.5 ± 4.3	48.7 ± 8.2	49.4 ± 3.1	48.4 ± 4.2	48.4 ± 8	45.6 ± 5	45.6 ± 4.4
	Varseosmile Crown^Plus^	45.9 ± 1.7	47.9 ± 4.8	45.9 ± 3.1	47.4 ± 3.8	48 ± 3.1	50 ± 4.4	45.9 ± 3	48.7 ± 5.6	49.5 ± 4.3
	Crowntec	106.7 ± 9.1	107.8 ± 12.8	111.1 ± 5.9	109.9 ± 8.5	105.5 ± 6.8	111.2 ± 7.9	99 ± 6.1	120.6 ± 8.1	102.7 ± 3.9
**Interproximal contacts (average mesial and distal surfaces)**										
**Material**	NextDent C&B MFH	34.5 ± 8.7	39.9 ± 7	44.6 ± 8.7	43.4 ± 8.6	48.5 ± 8.6	46.6 ± 12.2	51.4 ± 10.8	40 ± 8.3	42 ± 16.9
	Varseosmile Crown^Plus^	29.4 ± 7.3	43.7 ± 16.1	56.9 ± 18	40.4 ± 12.3	50.5 ± 10.8	54.1 ± 12.3	36.7 ± 7	38.4 ± 17.4	46.9 ± 9.1
	Crowntec	74 ± 8	76.5 ± 9.8	80.1 ± 7.8	91.5 ± 11.7	77 ± 10.2	75.6 ± 12.8	72.1 ± 6.9	109.2 ± 20.2	72.1 ± 5.9

RMS: root mean square; SD: standard deviation.

Conversely, when only using UV light, the hybrid resin composite (Varseosmile Crown^plus^) crowns (AM-VS groups) had the highest RMS values in intaglio and marginal surfaces after post-polymerizing in KZ (37.6 ± 5 μm) and ID (50 ± 4.4 μm) units, respectively. Utilizing the glycerine during post-polymerization led to a reduction in the RMS values of both intaglio and marginal surfaces, regardless of the polymerization units used (Table 4 and Figure 3). There were no statistically significant differences in the use of different post-polymerizing conditions for the marginal surface (Figure 3).

Compared to AM-ND and AM-VS materials, Crowntec crowns (AM-CT) exhibited the highest RMS values, with significant differences (*p* < 0.0001) (Table 4 and Figure 4). This is highlighted in the color map, with the majority of the examples showing negative deviations on the intaglio and marginal surfaces (Figures 6–8). For both intaglio and marginal surfaces of AM-CT groups and among all the polymerization conditions, the OF unit resulted in the significantly lowest RMS value when glycerine was used (Table 4). The different conditions in the OF unit caused significant differences in the AM-CT groups (Figure 3).

### Dimensional accuracy of interproximal contacts

There were significant differences in the mean RMS values for interproximal contacts among the SM (control group), AM-ND, and AM-VS crowns; however, the AM-CT group exhibited significantly higher RMS values than the other three groups (Figures 4 and 6–8). In both the AM-ND and AM-VS groups, the lowest RMS values were noted when the crowns were polymerized in the KZ unit with glycerine (Table 4). The AM-CT crowns had the lowest RMS values for interproximal contact areas using either glycerine or nitrogen in the OF polymerization unit. In contrast, when these crowns were polymerized using UV light in the OF unit, the highest RMS value was reported, showing significant differences from other post-polymerization units and conditions (*p* < 0.05).

### Positive and negative average deviation

In terms of the crown materials, the AM-CT crowns exhibited a significantly negative deviation in all analyzed areas (i.e. internal and marginal surfaces, and interproximal contacts) (Figure 4B). The color deviation map aligned with numerical results (Figures 6–8). Only the internal surfaces of AM-VS crowns differed significantly from the SM (*p* = 0.0303) and AM-ND (*p* = 0.0089) groups; otherwise, differences between the three materials were not significant (Figure 4B).

The average deviations of AM crowns after post-polymerization methods were inconsistent with RMS values (Tables 4 and 5). Marginal surfaces and interproximal contacts in all groups showed negative deviation, reflected by green-blue regions in the color deviation map (Figures 6–8).

AM-CT crowns showed negative deviations, evident by the dominance of the blue in color maps (Figures 6–8, Table 5). The highest negative deviation (−70.3 μm) occurred with UV in ND and OF units, while glycerine in OF produced the highest positive deviation. AM-VS crowns consistently showed positive deviations, with minimal surface changes and green/yellow-orange coloration. Crowns polymerized in glycerine with LC had the lowest positive deviation (2.8 ± 4.9 µm). For AM-ND crowns, the greatest positive deviation (10.1 ± 10 μm) occurred with nitrogen in OF, while glycerine in OF resulted in the lowest (−3.8 ± 8 μm).

### SEM analysis

**Figure 5:**
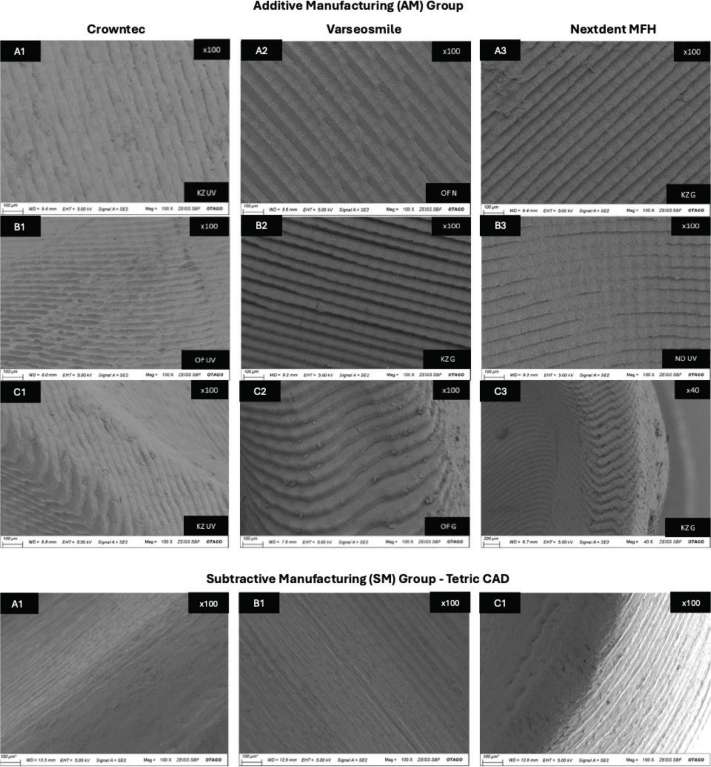
The SEM images of various surfaces of the materials used in the experiment. With group A showing the flat internal surface, group B showing the internal axial wall, and group C showing the marginal area of the crown.

SEM images of SM and AM crowns were captured to visualize the effects of different post-polymerization methods on the crowns surface (Figure 5). The surfaces selected for this analysis were the internal surface, internal axial wall and margin as these regions would not have any post printing polishing performed prior to the crown being cemented. Within group A, the print layers of Crowntec crowns appeared less distinct compared to those in NextDent and Varseosmile crowns. Varseosmile crowns displayed micro-cracks between print layers in groups A2 and C2. Group B shows the surface of the crown axial wall, where group B3 exhibited irregular print layers, suggesting incomplete cleaning of residual resin. Group B4 displayed thin, long lines indicating the path of the bur, while group A4 had rougher lines. The marginal area of the SM crowns (Figure 5C4) demonstrated a well-defined margin line with fewer bur marks compared to groups C1, C2, and C3, which showed more rounded edges.

## Discussion

This study investigated the effects of three post-polymerization conditions and four post-polymerization units on the dimensional accuracy of AM dental crowns. The findings indicate that post-polymerization temperatures (20°C vs. 60°C) did not significantly impact accuracy, though post-polymerizing in glycerine showed improved dimensional accuracy within certain material groups compared to UV only. Therefore, the hypothesis that post-polymerization conditions and temperatures would influence crown accuracy was partially accepted.

The dimensional accuracy varied depending on the surface being measured, with the intaglio surfaces generally showing lower RMS values than the marginal surfaces. This difference may be attributed to the larger surface area of the intaglio surface and more complex geometry, which likely introduces more variability in RMS values, while the smaller marginal surface displayed less statistical significance due to its limited area. Outcomes also varied by material type and post-polymerization methods. In the NextDent group, crowns polymerized in glycerine using the ND unit showed the best dimensional accuracy across all surfaces. This method yielded marginally lower RMS results for intaglio and marginal surfaces but was not significantly different from following the manufacturer’s recommendation of UV polymerization in the ND unit. For Varseosmile, both recommended methods (UV in ND unit and nitrogen in the OF unit) provided similar RMS values. Post-polymerising in glycerine using the KZ unit achieved a better overall dimensional accuracy, but significantly lower intaglio surface accuracy compared to the manufacturer’s IFU. For the Crowntec material, the recommended nitrogen atmosphere polymerization with the OF unit resulted in lower accuracy compared to the use of glycerine in the same unit, though the difference was not statistically significant. Notably, UV post-polymerizing in the OF unit for Crowntec yielded the highest RMS values across all surfaces. All RMS values for crown surfaces were within the clinically acceptable range (100 µm–120 µm), indicating that the manufacturer’s IFU and alternative post-polymerization methods could be viable. The discrepancies between mean positive/negative deviations and RMS are expected because RMS reflects magnitude irrespective of direction; over- and under-printing can yield small directional means while maintaining a large RMS values. Interpretation of the color deviation maps alongside RMS is beneficial as bidirectional deviations are visually evident allowing greater clinically relevant interpretation. Reporting both the RMS and positive-negative deviations is beneficial as it can illustrate the contraction (negative values) or expansion (positive values) of the material, which is clinically relevant to note especially for margin and interproximal area.

This study used a SM PMMA material for comparison as is a accepted dental standard due to its dimension stability [19], which enable AM technology’s to be compared for clinical viability. The intaglio and marginal surfaces of SM crowns showed the lowest mean RMS values compared to AM crowns. However, the interproximal contacts of SM crowns exhibited RMS values similar to AM crowns. This similarity may stem from the complexity of the interproximal geometry, where tool limitations restrict precise replication of the surface structure. Observed deviations suggest that the crown geometry contracts cause marginal shrinkage and intaglio surface compression. This could explain the positive average deviations in intaglio surfaces and negative deviations in marginal surfaces (Tables 3 and 4).

Within the NextDent group, post-polymerization conditions and units had no significant effect on intaglio and marginal surface RMS values, indicating the material’s versatility to various post-polymerization conditions. This may be due to the material’s composition, including acrylic oligomers, microfillers, and inorganic fillers, which could have minimized distortion of polymer chains during post-polymerization. Cornelio et al. [20] observed that the lower viscosity of bis-EMA contributes to a gradual cross-linking process, potentially increasing polymer chain length [21]. Crowntec contains 50–70% bis-EMA (Table 1) and demonstrated a lower dimensional accuracy, likely due to its lower initial viscosity and slower cross-linking speed. However, this explanation assumes that composite and AM dental crown resins are directly comparable.

The different cleaning protocols between Crowntec and other materials also likely contributed to variability. The manufacturer’s IFU recommended manual alcohol-brush cleaning could have introduced inconsistencies due to human error and a lack of standardization, potentially resulting in rougher print layers observed under SEM (Figure 5). The manufacturer may have recommended this protocol to reduce alcohol degradation and improve mechanical properties, however the surface of the crowns seem to exhibit uncured photopolymer after the post washing procedure, which may lead to variability in the surface. The generally favorable performance under inert media is consistent with reduced oxygen-inhibition at the surface, which may limit the post-curing of the surface; this offers a plausible basis for the lower RMS observed in several glycerine groups. From a clinical and technical perspective, adopting an inert medium during post-curing process is a simple intervention that may improve dimensional stability and accuracy without major workflow changes.

One limitation of this study was the inability to capture SEM images of the interproximal surfaces due to time constraints, which restricted the understanding of trends in interproximal contact graphs. Given that these surfaces would be polishing prior to insertion this may not be a clinically relevant approach to investigation. Another limitation was the 45-degree printing angle, which may have increased error in crown thickness [22], contributing to positive deviations in AM materials (Figure 4). The new cleaning concentrate of Saremco material was not investigated by this study, but this may have helped to clean the specimens equally. The different light intensities of each post-polymerization unit could have also influenced the results [23]. In addition, the increase of the post-polymerization duration for Varseosmile – from 1,500 flashes x2 to 2,000 flashes x2 in the Otoflash unit may have influenced the RMS values as it deviated from the manufacturer’s recommended settings.

Given the variations in AM resins and the diverse properties, its post-processing protocol demands meticulous attention. Small adjustments can significantly influence material characteristics, underscoring the need for optimized post-polymerization protocols that reinforce strength without compromising dimensional accuracy. Future studies should focus on refining these protocols to ensure the material’s long-term reliability and clinical viability.

## Conclusion

Within the limitations of the study, the following conclusions can be drawn:

1. Submerging crowns in glycerine with the manufacturer’s recommended post-polymerization unit generally resulted in better dimensional accuracy.
2. Post-polymerization units that function at 20°C did not significantly impact results when compared to units that function at 60°C.
3. The subtractive manufactured crowns showed the highest dimensional accuracy for the intaglio and marginal surfaces, whereas the RMS values for the interproximal contacts did not differ significantly.
4. The average deviation results may suggest that the entire geometry of the crown experiences contraction, shrinking the margin as a result of the compression within the intaglio surface.
5. Some SaremcoCrowntec RMS values were higher than the clinically acceptable range of 100–120 μm for certain post-polymerization methods.
